# A Novel Approach for UAV Image Crack Detection

**DOI:** 10.3390/s22093305

**Published:** 2022-04-26

**Authors:** Yanxiang Li, Jinming Ma, Ziyu Zhao, Gang Shi

**Affiliations:** College of Information Science and Engineering, Xinjiang University, Urumqi 830046, China; liyanxiang@stu.xju.edu.cn (Y.L.); majinming@stu.xju.edu.cn (J.M.); 107551901060@stu.xju.edu.cn (Z.Z.)

**Keywords:** crack detection, deeep learning, target detection, image stitching, unmanned aerial vehicle

## Abstract

Cracks are the most significant pre-disaster of a road, and are also important indicators for evaluating the damage level of a road. At present, road crack detection mainly depends on manual detection and road detection vehicles, with which the safety of detection workers is not guaranteed and the detection efficiency is low. A road detection vehicle can speed up the efficiency to a certain extent, but the automation level is low and it is easy to block the traffic. Unmanned Aerial Vehicles (UAV) have the characteristics of low energy consumption and easy control. If UAV technology can be applied to road crack detection, it will greatly improve the detection efficiency and produce huge economic benefits. In order to find a way to apply UAV to road crack detection, we developed a new technique for road crack detection based on UAV pictures, called DenxiDeepCrack, which is a trainable deep convolutional neural network for automatic crack detection which utilises learning high-level features for crack representation. In addition, we create a new dataset based on drone images called UCrack 11 to enrich the crack database of drone images for future crack detection research.

## 1. Introduction

Annually, millions of USD are spent in the process of crack detection on roads. Roads suffer from long-term damage such as being crushed by vehicles, temperature changes or natural disasters. These damages may lead to cracks in the road. These cracks make the roads’ load-bearing capacity weaker and lead to surface discontinuities. The cracks may spread across the surface and may lead to complete collapse of the structure. The damage can be reduced if cracks can be detected at an early stage. The manual method is still the mainstream detection method, which is tedious, laborious, does not guarantee the safety of personnel, and is particularly dependent on the experience of experts. Therefore, experts and scholars have proposed some automated methods. Methods of obtaining crack information can be divided into laser, infrared, thermal, radiographic and thermal testing methods.

Recently, image-based crack detection has received increasing attention in nondestructive testing. The main advantage of image-based crack detection is that it provides accurate results compared to traditional manual methods by using image-processing methods [1]. The high quality and multidisciplinary nature of the images from UAV cameras, and the feature that UAVs can go to places that are difficult to photograph because of their flexibility, have been applied to bridge and building crack detection. Compared to other methods such as inspection vehicles, drones are able to obtain information about road cracks in a more convenient way and without affecting road traffic. With low energy consumption, low cost, and low pollution, using drones in road crack detection could provide greater economic benefits.

In traditional image-processing methods, various tasks need to be conducted to achieve noise removal on the image, because noise can have a great impact on the algorithm. As for deep learning, it is not necessary to remove the noise because the algorithm itself can discriminate the noise. However, the network needs to learn the features of the noise before it can remove the noise.

In this paper, two approaches were used to improve noise recognition ability. First, image enhancement is used for the dataset and noise samples are added, and then DeepaCrack is improved based on the idea of Dense Extreme Inception Network (DexiNed) [2] to make the extracted features more accurate and robust. Many works have produced various image-based datasets [3,4,5,6]. The creation of these datasets has accelerated the development of crack detection, but there is no UAV-based dataset. So, this paper uses the collected images by UAV and manually labels a dataset, UCrack.

The contributions of this work are as follows:

1. The problem of multiple images with duplicate regions and the problem of images with occlusion are proposed for the first time, and an innovative way of combining target detection and image stitching is used to deal with these two problems.
2. A DenxiDeepCrack algorithm is proposed and experimentally demonstrated to be superior in crack detection based on UAV images.
3. To be able to apply drone technology to crack detection, we manually labeled a dataset based on drone road pictures of cracks.

The rest of this paper is organized as follows. Section 3 describes the methods of this work, and introduces vehicle detection, feature-based image stitching and pixel-level crack detection in detail. Section 4 describes the experimental details of each part. Section 5 concludes the paper.

## 2. Background and Related Works

The performance of crack-detection algorithms will directly determine the results of image-based pavement crack detection. Crack-detection algorithms can be divided into those based on traditional image-processing techniques as well as those based on deep-learning techniques. Early recognition algorithms are based on traditional digital image-processing algorithms [7], such as threshold segmentation, feature extraction, edge detection, filters, and minimum path methods.

Li et al. [8] used an adjacent difference histogram method to identify regions containing cracks, assuming that pixels belonging to the cracked region are always darker than other pixels. Li et al. [9] used twice-thresholding and adaptive iterative thresholding to detect cracks on airport runway surfaces. Kapela et al. [10] used Hough transform feature (HTF) and local binary pattern (LBP) to extract the edge orientation and texture features of cracks, respectively. Abdel-Qader et al. used four edge-detection methods, namely Canny edge detector, Sobel edge detector, fast Fourier transform and fast Haar transform, to detect concrete cracks [11], and they found FHT to be the best solution.

Based on minimum path localization, Amhaz et al. [12] proposed an automatic detection algorithm to detect 2D pavement cracks. All crack-detection algorithms based on conventional digital image processing perform well on datasets with discriminative and representative depth features. However, due to the complexity of real pavement conditions and various uncertainties in terms of environmental influences, such as texture diversity, strong noise interference, and irregular crack orientation, these algorithms are vulnerable to environmental factors and cannot meet the needs of both accuracy and speed.

In recent years, deep convolutional neural networks (DCNN) have proven to be advanced, human-competitive, and sometimes even better than human performance in solving many computer vision problems, such as image recognition [13], object detection [14], and semantic image segmentation [15]. Recently, scholars have proposed a series of neural network-based crack detection and recognition algorithms for cracks in different situations. Eisenbach et al. [16] proposed a road disease dataset for training deep-learning networks and provided the first evaluation of the state of the art in pavement disease detection. Zhang et al. [17] classified convolutional neural networks for fractured and non-fractured plates and demonstrated the advantages of deep learning in fracture detection. The neural network could classify fractured and non-fractured plates and demonstrated the advantages of deep learning in fracture detection. Li et al. [18] proposed a convolutional encoder–decoder network (CedNet) to detect cracks from images, and created a dataset including 1800 crack images (with 761 × 569 pixel resolution) taken from concrete structures. Xu et al. [19] proposed a joint training strategy with Faster R-CNN and Mask R-CNN, which can achieve good results than YOLOv3 with little training images. Fan et al. [20] proposed a supervised method based on deep learning. The method provides good detection of pavement cracks in different environments by modifying the positive-to-negative ratio of the samples. In 2020, Chen et al. [21] proposed a rotation-invariant FCN called ARF-Crack that utilizes the rotation-invariant property of cracks explicitly. The proposed ARF-Crack requires a smaller number of network parameters. Nhung et al. [22] proposed a method that utilises a two-stage convolutional neural network for road crack detection and segmentation in images at the pixel level. Aravindkumar et al. [23] present a novel, multi-tasking, Faster-RCNN-based approach using the Global Average Pooling (GAP) and Region of Interest (RoI) Align techniques to detect road cracks. Qin et al. proposed a method to fuse convolutional features based on SegNet [24] encoder–decoder network to build a new DeepCrack [25] network for crack detection, and achieved the state-of-the-art results.

Previous studies were based on single images, and they did not discuss how to deal with duplicate areas of images. Regardless of using handheld cameras or in-vehicle cameras, duplicate regions are inevitably present in images. If the duplicate region is not removed, then the detected result will be impacted. Because the UAV is shooting in the air, there will be vehicles and other occlusions in the captured images.

In this paper, an innovative combination of image stitching and target detection is proposed to solve these two problems. In addition, images captured by the UAV have fewer crack pixels compared to those taken at close range, and are more prone to the all-black phenomenon during the detection process [26]. In this paper, we adopt migration learning to successfully solve this problem. According to the article illustrated in [27,28], noise can seriously affect the detection results, and every study has made a lot of effort to removie noise.

## 3. Proposed Approach

**Figure 1:**
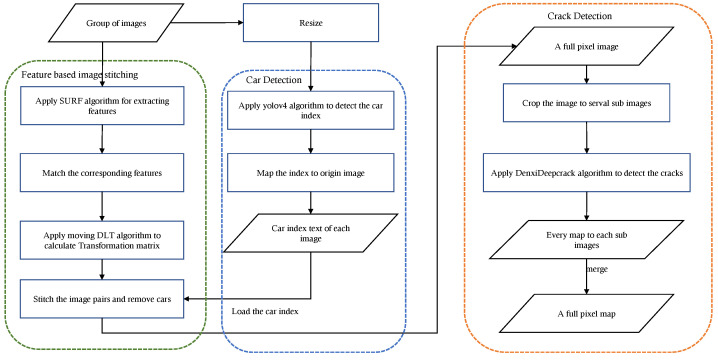
Flowchart of the proposed method.

The proposed method in this work has three main components. (i) vehicle detection, (ii) feature-based image stitching, and (iii) crack detection. Figure 1 shows the flow of this method. First, because the target-detection algorithm is able to detect the location of the target object, it is applied to vehicle detection, as shown in the center part of Figure 1. Then, the Speeded Up Robust Features (SURF) algorithm is used to extract the features in the images and to match the features in different images. The matched features are used to calculate the transfer matrix using the moving DLT algorithm. Then, the vehicles are removed during the final stitching process based on the obtained vehicle location information. Finally, the crack-detection algorithm is used to detect the cracks in chunks of the stitched large image and merges them to generate the complete map.

### 3.1. Vehicle Detection

#### 3.1.1. YOLOv4 Model

For vehicle detection, due to optimal speed and accuracy of YOLOv4, it is used in target detection. The task of target detection is to find all the targets of interest in an image and determine their class and location. Target detection is one of the core problems in the field of computer vision. There are two major tasks in target detection, which are classification (what is it), and localization (where is it). Deep-learning-based target-detection algorithms are mainly divided into two categories, two-stage and one-stage. The two-stage category is called region proposal (RP, a pre-selected box that may contain the object to be detected), where the target candidate region is first selected for the input image, and then the extracted features are classified and position-regressed. In one-stage algorithms, features are extracted directly in the network to predict object classes and locations. Two-stage target-detection methods first generate a large number of a priori boxes that may contain the object to be detected, then use a classifier to determine whether the bounding box corresponding to each a priori box contains the object to be detected and the probability or confidence of the object’s class, and also requires post-processing to correct the bounding boxes, and finally filters out the bounding boxes with low confidence and high overlap based on some criteria to obtain the detection results. This approach, based on generating candidate regions and then detecting them, has relatively high detection accuracy, but it is slow.

**Figure 2:**
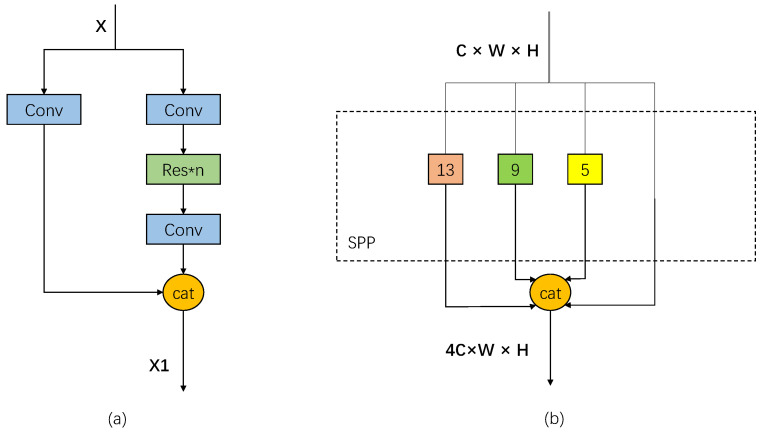
Structure of CSP and SPP. (**a**) CSP. (**b**) SPP.

YOLO creatively treats the object-detection task directly as a regression problem, combining the candidate and detection phases into one. In fact, YOLO does not really remove the candidate regions, but uses predefined anchors. After many iterations of the YOLO series, YOLOv4 achieves optimal speed and accuracy. YOLOv4 inherits the backbone structure of YOLOv3 [29], and in the feature-extraction structure, unlike the continuous stacking of residual blocks inside DarkNet53 in YOLOv3, it incorporates the CSPNet structure and proposes CSPDarkNet53, which splits the stacked residual blocks into two parts, the backbone continues the stacking of the original residual blocks, and the other part is directly connected to the end after a small amount of processing like a residual edge. Figure 2a shows one of the stacked residual blocks using the Cross Stage Partial (CSP) structure. X represents the input feature map. Conv represents one convolution operation. cat indicates the operation of stacking in the channel dimension in the structure of feature fusion. SPP (Spatial Pyramid Pooling) is used to increase the perceptual field. As shown in Figure 2b, C, W, and H represent the number of channels, width and height of the feature map, respectively. The maximum pooling is processed with five different sizes of maximum pooling kernels of 13 × 13, 9 × 9, 5 × 5 and 1 × 1, respectively, replacing the Feature Pyramid Networks (FPN) structure in YOLOv3 with PANet, which can better fuse the features by fusing the features from high to low and then fusing them from low to high again.

#### 3.1.2. Loss

For one of anchors, the loss function of YOLOv4 is shown in Equation (1):

(1) $$L=L_{reg}+L_{conf}+L_{cls}$$

The loss function consists of three main components, which are the bounding box regression loss $L_{reg}$, confidence loss $L_{conf}$ and classification loss $L_{cls}$. For the bounding box regression loss, YOLOv4 [30] replaces the traditional Mean Squard Error (MSE) function with the Complete-Intersection Over Union (CIOU) function, which enables the bounding box to move faster towards the true one. The confidence loss function is a cross-entropy function, which calculates the confidence loss regardless of whether the anchor generates a prediction box containing an object or not. The classification loss function is also a cross-entropy function, which calculates the confidence loss when the anchor is responsible for the true object prediction. The classification loss function is also a cross-entropy function, and the classification loss is calculated for the prediction frame generated by the anchor only when the anchor is responsible for the real object prediction.

### 3.2. Feature-Based Image Stitching

#### 3.2.1. Speeded Up Robust Features

The keys to obtaining a seamless high-resolution image are image alignment and image stitching. Scale-Invariant Feature Transform (SIFT) [31] and Speeded Up Robust Features (SURF) [32] are the most popular methods used to extract features. In this paper, the SURF algorithm is used because SURF [33] has a lower dimensionality than SIFT, and therefore it is faster and more suitable for a task that requires real-time processing.

#### 3.2.2. Mathematical Setup

Let *I* and $I^{\prime }$ denote the target image and the reference image, respectively. The homography warp *H* is a planar transformation and a 3 × 3 matrix. It relates the pixel coordinates (x,y)∈I to $\left(x^{\prime },y^{\prime }\right)\in I^{\prime }$ by

(2) $$\left\{\begin{array}{cc} x^{\prime }=f\left(x,y\right) & \\ y^{\prime }=g\left(x,y\right), \end{array}\right.$$

where

(3) $$f\left(x,y\right)=\frac{h_{1}x+h_{2}y+h_{3}}{h_{7}x+h_{8}y+1},g\left(x,y\right)=\frac{h_{4}x+h_{5}y+h_{6}}{h_{7}x+h_{8}y+1}.$$

and

(4) $$H=\left[\begin{array}{ccc} h_{1} & h_{2} & h_{3}\\ h_{4} & h_{5} & h_{6}\\ h_{7} & h_{8} & h_{9} \end{array}\right]$$

Traditionally, *H* is estimated from a set of feature correspondences by direct linear transformation (DLT). If there is a parallax between different views, then *H* does not align the images well because the motion and scene assumptions are not satisfied. In contrast to using a single global homologous warp to align images, APAP [34] uses a spatially varying warp H*, consisting of multiple local homologous warps for image alignment. In other words, APAP assigns position-dependent weights to feature correspondences, which allows for local fine-tuning of homography that assigns the same weights to feature correspondences, resulting in better alignment of overlapping regions. This approach is known as moving DLT, and it has proven to be very effective for image alignment, especially in the case of general motion and non-planar scenes.

**Figure 3:**
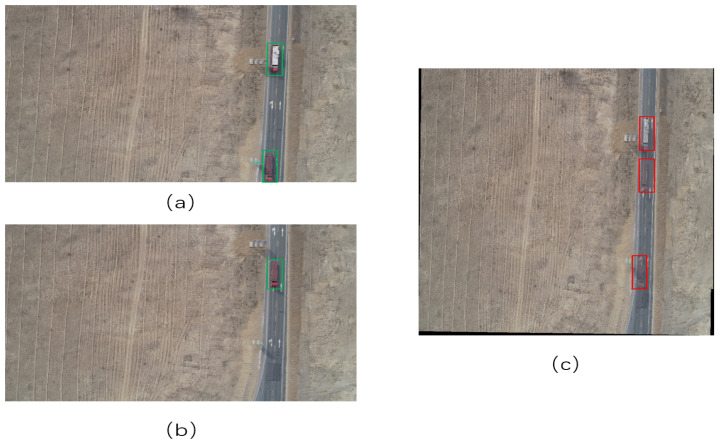
The two stitched images using Equation (4). (**a**) Image to be stitched with trucks, (**b**) another image to be stitched with a truck, (**c**) the result of (**a**,**b**) to be stitched by Equation (5).

Normally two pixels that matched to one pixel would be stitched together by

(5) $$p_{i}=p_{ori}\times 0.5+p_{ref}\times 0.5,$$

where $p_{ori}$ represents the pixel point in the original image and $p_{ref}$ is the pixel point of the image to be stitched. However, if we use Equation (4) to stitch images, the stitching result will be as shown in Figure 3. There are two trucks in Figure 3a (green box) and Figure 3b has one truck (green box). As you can see in Figure 3c, there are three trucks (red box). All of these trucks were retained. Therefore, for the pixels at the position of the vehicle, we use the following stitching method:

(6) $$p_{i}=p_{ins},$$

where $p_{ins}$ represents the pixel value of the part of the other image that does not have a vehicle at the corresponding position.

### 3.3. Crack Detection

#### 3.3.1. DenxiDeepCrack

**Figure 4:**
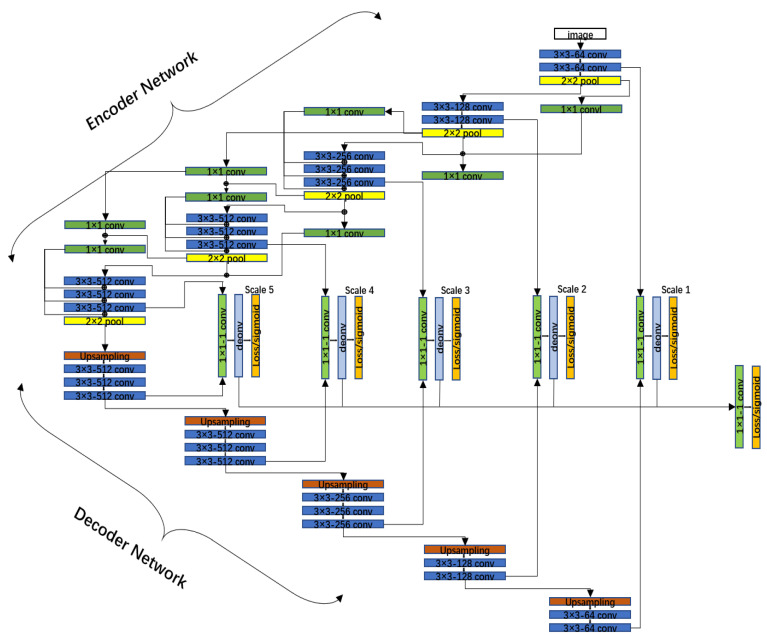
Architecture of the DenxiDeepCrack model.

Cracks, in practice, have poor continuity and low contrast. DeepCrack is an end-to-end, trainable, deep convolutional neural network for automatic crack detection which works by learning high-level features for crack representation. In this approach, multi-scale deep convolutional features learned in the hierarchical convolution stage are fused to capture the crack structure. By this approach, we can partly solve this problem. Unlike handheld or vehicle-mounted cameras, UAV capture cracks at a greater distance from the phase hole point, which means that more feature information is needed for better utilization of this feature information. Therefore, we designed the DenxiDeepCrack network. It consists of five encoders and five decoders. It is inspired by the Dense Extreme Inception Network (DexiNed) [2], changing the near-symmetric network of DeepCrack. Since multiple convolutions are performed and important edge features are lost in each depth block, one main connection alone is not enough, so the output of each sub-block is averaged with edge connections starting from block 3 (orange square). As shown in Figure 4, after the max-pool operation and before the main connection summation, the edge connections are set to average the output of each sub-block (see the green rectangle, bottom). From the max-pool, the layer 2 edge connections provide information for layers 3, 4 and 5.

#### 3.3.2. Loss

Given a training data set containing *N* images as $S=\{\left(X^{n},Y^{n}\right),n=1,\ldots ,N\}$, where $X^{n}=\left\{x_{i}^{\left(n\right)},i=1,\ldots ,I\right\}$ denotes the raw input image, $Y^{n}=\left\{y_{i}^{\left(n\right)},i=1,\ldots ,I,y_{i}^{\left(n\right)}\in \left\{0,1\right\}\right\}$ denotes the ground-truth crack label map corresponding to $X^{n}$, and *I* denotes the number of pixels in every image, our goal is to train the network to produce prediction maps approaching the ground truth. In the encoder–decoder architecture, let *K* be the number of convolution stages, then at the stage *k*, the feature map generated by the skip-layer fusion can be formulated as $F^{\left(k\right)}=\left\{f_{i}^{\left(k\right)},i=1,\ldots ,I\right\}$, where k=1,…,K. Further, the multi-scale fusion map can be defined as $F^{fuse}=\left\{f_{i}^{fuse},i=1,\ldots ,I\right\}$.

There are only two categories in crack detection, which can be regarded as a binary classification problem. We use cross-entropy loss to measure the prediction error. The prediction loss within the pixel range is:

(7) $$l\left(F_{i};W\right)=\left\{\begin{array}{cc} log(1-P\left(F_{i};W\right)), & if\;\;y_{i}=0,\\ log(P\left(F_{i};W\right)), & otherwise, \end{array}\right.$$

where $F_{i}$ is the output feature map of the network at pixel *i*, *W* is the standard set of parameters of the network layer, and P(F) is the standard sigmoid function that transforms the feature map into a crack probability map. Then, the total loss can be expressed as:

(8) $$L\left(W\right)=\sum_{i=1}^{I}\left(\sum_{k=1}^{K}l\left(F_{i}^{\left(k\right)};W\right)+l\left(F_{i}^{fuse};W\right)\right).$$

### 3.4. UCrack

Using a DJI Phantom 4 drone, we chose a section of the highway in Xinjiang, China, and flew a total of six kilometers back and forth along the centerline at a 90° angle, in other words, vertically down. We took one picture per second and ended up with 876 8192 × 5460 UHD images. We selected 100 of these images with more crack samples for annotation. The image-labeling tool is labelme [35], developed by MIT’s Computer Science and Artificial Intelligence Laboratory (CSAIL). It does not allow for polygon fitting because of the narrow crack pixels. The method we use is to draw a line to fit this crack, and each crack is recorded as a linestrip and stored as one of the objects in json format. Each image corresponds to a json file, and these json files store the coordinates of the point corresponding to each crack in the image. Finally, using opencv’s generation curve, we use these point coordinates to adjust the width of the generated curve and obtain the map corresponding to each image.

## 4. Experimental Results

In this paper, experiments are conducted to verify the proposed method, and three aspects of vehicle detection, image stitching, and finally crack detection are demonstrated.

### 4.1. Vehicle Detection

The target-detection algorithm and image stitching used in this paper are actually pre-processing for crack detection, and the focus of our work is crack detection. Therefore, no comparison is made between this algorithm and other algorithms, and only experimental details as well as experimental results are given.

#### 4.1.1. Dataset

The VisDrone2019 [36] dataset was collected by the AISKYEYE team at the Lab of Machine Learning and Data Mining, Tianjin University, China. The benchmark dataset consists of 288 video clips comprising 261,908 frames and 10,209 images captured by various UAV-mounted cameras covering a wide range of aspects including location (taken from 14 different cities in China separated by thousands of kilometers), environment (urban and rural), objects (pedestrians, vehicles, bicycles, etc.), and density (sparse and crowded scenes). The frames are manually annotated by bounding boxes of more than 2.6 million frequent targets of interest, such as pedestrians, cars, bicycles and tricycles. To better utilize the data, some important attributes are also provided, including scene visibility, object class, and occlusion. Considering that bicycles and various modes of transportation such as pedestrians are likely to be present on the road, the full dataset is used for this work. We divided the 6471 images in the dataset into a training set as well as a validation set in the ratio of 8 to 2. The number of targets in the training and validation sets are as in Table 1.

#### 4.1.2. Training

Experiments were carried out based on the deep-learning framework of Darknet platform on a computer with E5-2690V4 CPU, NVidia GeForce GTX 2080Ti 11 GB GPU and 16 GB of memory, running on a Ubuntu 16.0 system. The software tools included CUDA 10.0, CUDNN 7.5, OpenCV 3.4.5, and Visual Studio 2017. Input size, batch size, learning rate, momentum, and iteration were set as shown in Table 2. Before training, we transferred the dataset to YOLO format and used K-means cluster method to define the sizes of the anchor boxes. We set k as 9, and after the experiment, the result showed 9 different sizes of anchor boxes, which were (14, 14), (19, 20), (23, 26), (30, 28), (30, 36), (38, 33), (41, 44), (57, 57), (86, 89), while the pixel size of the image was fixed to 416 × 416.

#### 4.1.3. Results

**Figure 5:**
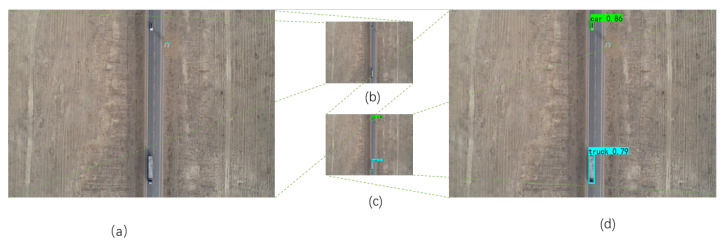
Vehicle Detection. (**a**) The original imge, (**b**) The resized image, (**c**) Resized image to be deteced, (**d**) Recovered to final result.

The previously mentioned YOLOv4 model trained with the visdrone dataset was detected on the 876 images we took. Because of the large pixel size of the images, the vehicle features are very obvious compared to the surrounding environment. Therefore, resizing the original images to 416 × 416 can not only improve the speed of detection, but also ensure the accuracy of detection. Figure 5a shows the original image to be detected on the left, with two vehicle occlusions on it. Figure 5b is the reduced image. Figure 5c is the result after the detection by YOLOv4 algorithm, and Figure 5d is the final result. Finally, the location information of the occluded vehicles is saved as a text for the next stage which is the image-stitching stage. Table 3 is a comparison of the results of 876 images manually counted with the YOLOv4 detection results; you can see the number of cars and vans, for which the detection results are one less and one more respectively. This is because one of the pictures only shows half of the van, so it is hard to tell whether it is a car or a van. However, the total number and location are correct, which means that the method is fully capable of detecting the location of the vehicle in the drone pictures.

### 4.2. Feature-Based Image Stitching

**Figure 6:**
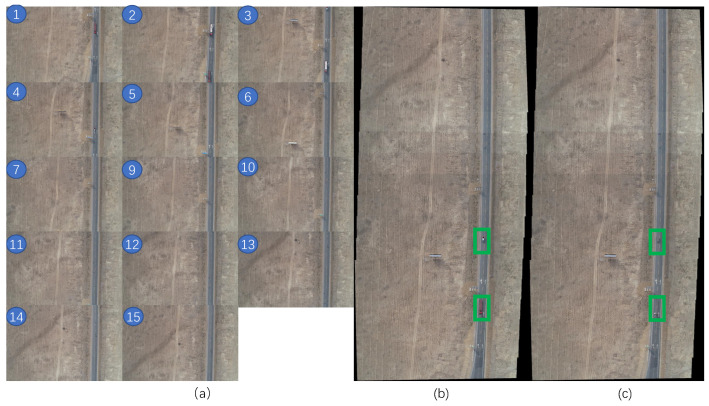
Image Stitching. (**a**) Images to be stitched, (**b**) stitched by Equations (5), (**c**) stitched by Equation (6). The number in the blue circle indicates the order of stitching.

As you can see from Figure 6a, the fifteen images are the objects that we will be stitching together. You can see that images 1, 2 and 3 all have vehicles above the road. Before stitching, we have already obtained the position of each vehicle for each image from the previous step. The stitching is performed in a recursive way, by first stitching two adjacent images together, and then stitching the stitched images together again in this way. For example, the first and second are stitched together as a, then the third and fourth are stitched together as b, and the image a and image b are spliced together and so on. In this way, it saves more time than sequential stitching. Figure 6b,c show the result without removing the vehicles and the result of removing the vehicle, respectively. As can be seen by the location marked by the green box, the vehicles obscure part of the highway, but after removal, the highway in that area is revealed. As shown in Figure 6c, The part of the vehicle that was removed is a bit unnatural. This is because the number of pixels in the image is so large that it is difficult for the image to be stitched exactly correctly. However, this degree of error is much more acceptable than the error caused by vehicle occlusion.

### 4.3. Pixel-Level Crack Detection

In this paper, three strategies are used to optimize the network training process, namely, using a migration learning strategy, adding noise information, and improving the DeepCrack network, and experiments are performed to verify the necessity of each strategy. To demonstrate the superiority of the methods used in this paper, state-of-the-art algorithms in the field of crack detection are selected for comparison.

#### 4.3.1. Dataset

CrackTree260 contains 260 road images and is an extension of the dataset used in [37]. These road images were captured by an area array camera under visible illumination. We used all 260 images for migration training. To expand the dataset, we used the same strategy as in [25]. We rotated the images at 9 different angles (from 0 to 90 degrees with an interval of 10 degrees), flipped the images vertically and horizontally at each angle, and cropped 5 sub-images (4 in the corners and 1 in the center) on each flipped image with a size of 512 × 512. After the expansion, we obtained a training set with a total of 35,100 images.

UCrack contains 100 images of our manually labeled drone roads. The 100 images are divided into a training part and a test part in the ratio of 8:2. Due to the large pixel size of the images and the limitation of GPU memory, we cropped the 80 8192 × 5460 images of the highway and some nearby areas for training into 512 × 512 images, and obtained 1786 images in total. Finally, the 1786 images were randomly divided into a training set and validation set according to a ratio of 9:1, in other words, into 1607 and 179 images respectively. The training dataset is called dataset${}_{noise}$. In order to perform a comparison, we obtain the road part of the above eighty images and crop them into 512 × 512 parts, and obtain 1556 images, 1401 of which are used as a training set and 155 are used as a validation set, named as dataset${}_{nonoise}$.

#### 4.3.2. Training

In this paper, there are two groups of comparison experiments. The first group is divided into three groups according to the training method used. The first group named A uses the dataset${}_{noise}$ directly as the training dataset without migration learning, the second group named B uses the migration learning method and dataset${}_{nonoise}$, and the third group named C uses migration learning and trains with the dataset${}_{noise}$. The detection algorithm used in all three approaches is DenxiDeepCrack.

The other group uses all the approaches mentioned above and then the detection algorithm is different. The six algorithms are DenxiDeepCrack, DeepCrack, Segnet, Fcn, Unet, and Resnet. In training, the initial global learning rate is set to 1 × 10^−5^ and divided by 10 after every 10 K iterations. The attenuation of momentum and weight is set to 0.9 and 0.0005. Both the migration learning and training processes have carried out 100 k iterations of all experiments in this paper using GenForce Gtx-2080Ti.

#### 4.3.3. Metrics

For each image, precision and recall can be calculated by comparing the detected cracks with the human-annotated image. Then, an F-score can be calculated as an overall metric for performance evaluation. Specifically, three different F-score-based metrics are used in the evaluation: fixed threshold (ODS), aggregated F-score on the optimal threshold on each image (OIS) dataset, and average precision (AP).

ODS (optimal dataset scale), also known as global best, fixed threshold i.e., fixed scale of the dataset, and optimal on the dataset scale of the detection metric, simply means setting the same threshold for all images, i.e., selecting a fixed threshold to be applied to all images so that the F-score on the entire dataset is maximized.

OIS (optimal image scale), also known as single-image optimal, is the optimal threshold for each image, that is, a different threshold is selected for each image that maximizes the F-score of that image.

AP (average precision) is the integral of the PR curve (i.e., the area under the PR curve). Since the PR curve is difficult to integrate, it is usually sampled on the PR curve to find the mean value.

#### 4.3.4. Results

**Figure 7:**
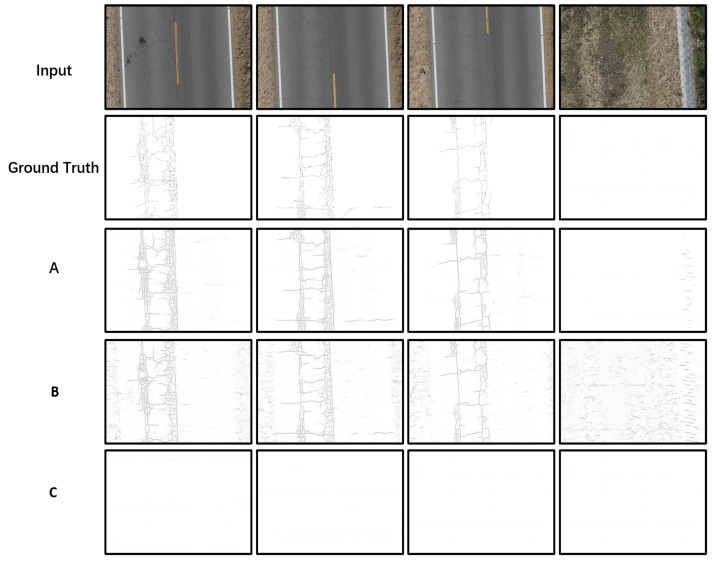
Results for the crack detection of three different methods on UCrack. (**A**) Group A named in Section 4.3.2, (**B**) Group B, (**C**) Group C.

Figure 7 shows the results obtained from three training methods. This is an arbitrarily selected graph of our results, and in order to be able to show it more clearly, we performed inversion and took parts of the image. You can see that without migration learning, the all-black phenomenon appears, resulting in none of the cracks being recognized (“all-white” due to the inversion). With the inclusion of migration learning, the model is particularly affected by noise if the only samples we take in the training set are the highway parts. As shown in Figure 7B, the cracks on the surface of the road are recognized well, but the parts on both sides of the road are also recognized as cracks, which is obviously impacted by noise. It can also be seen from the Table 4 that due to the appearance of the all-black phenomenon, each value is 0 and not a single crack is detected. The group without adding noise has very low values, even the AP value is only 0.073, because most of the cracks identified are wrong and the noise counts for a particularly large part of the image. The third method has far better results than the first two. This shows that both of the data-enhancement methods used in this paper are very effective.

**Figure 8:**
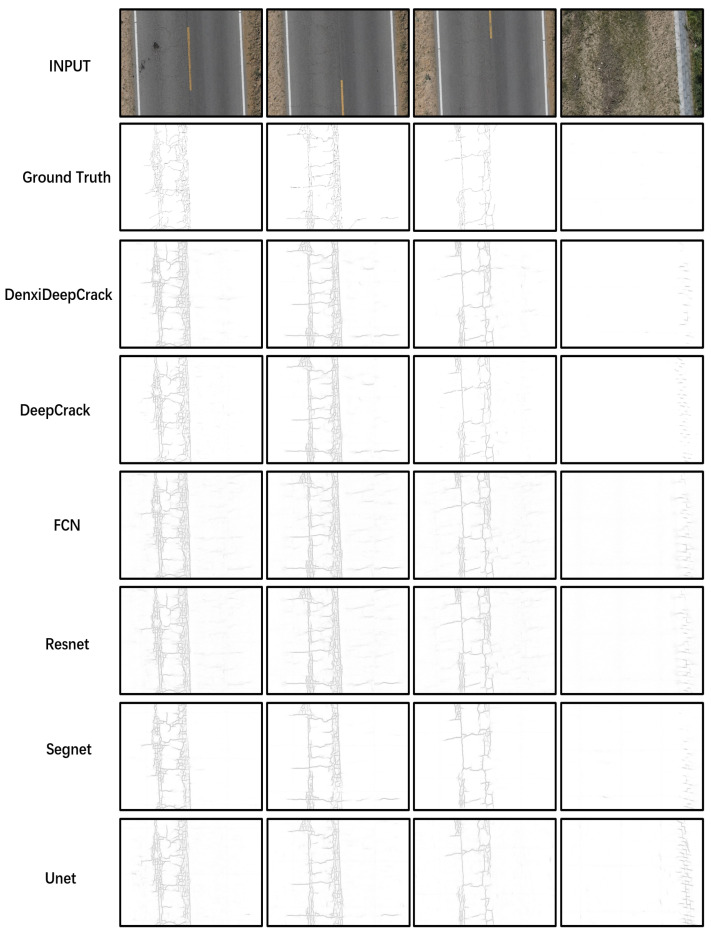
Results of the six algorithms on the UCrack.

Figure 8 shows the results of the six algorithms on the UCrack dataset. It can be seen that the encoder–decoder network structures such as Segnet, Unet, DeepCrack, and DenxiDeepCrack are more accurate for crack identification. The case that the right side of the road is misidentified as a crack rarely exists. The image on the right corner has a very strong noise, and by comparison, it can be seen that the DenxiDeepCrack algorithm has the best ability to handle this noise. In part of road, DenxiDeepCrack identifies more complete cracks with better details, closer to the manually labeled map.

As shown in Table 5 DenxiDeepCrack achieves the best results. Each of these metrics is 10 better than the next best. In comparison, DeepCrack fuses the multi-scale convolutional features in both the encoder and decoder networks. The effect is not very significant. It is only a little higher than segnet. However, Resnet, which uses ideas such as the Densenet mechanism, is much higher than Fcn, so the idea of connecting the layers and fusing features with each other is particularly effective. In summary, DenxiDeepCrack achieves the best results on UCrack.

## 5. Conclusions

In this work, we propose a novel approach to detect cracks, called DenxiDeepCrack, using deep-learning models. We create a dataset based on drone images called UCrack. To validate the proposed approach, We conducted experiments using road images taken by a UAV. The obtained images first acquire vehicle location information after detection by the YOLOv4 algorithm. Afterward, the obtained vehicle information is used to remove the vehicles in the image stitching process, creating a large image with no occlusion information and duplicate parts removed. Next, the DenxiDeepCrack algorithm is used to detect the cracks in the image.

To make the detected cracks more accurate, two training strategies are used. First, the CrackTree260 dataset is used as migration learning, which solves the all-black phenomenon that occurs during the detection process. After that, the labeled map is filtered, and a few pixels near the highway and the highway are selected. This solves the problem of excessive noise. According to the results, we successfully removed the occlusion and repetition of the UAV image. The algorithm DenxiDeepCracak had good results.

The crack-detection algorithm used in this paper does not classify the types of cracks. In the actual application scenario, it is necessary to first determine the cracks and non-cracks, and also the different types of cracks, and this aspect is not studied in this paper. This is the direction of our future research on the process of crack detection.

## Figures and Tables

**Table 1:** Nums of different target in VisDrone2019.

Dataset	Num	Car	People	Van	Truck	Motor	Bicycle	Tricycle	Awning-Tricycle	Bus
Training dataset	5176	115,895	22,441	17,470	10,944	23,717	7860	4186	2565	4623
Validation dataset	1295	28,972	5500	7486	1931	5930	2620	626	681	1303

**Table 2:** Network parameters of YOLOv4.

Input	Batch Size	Learning Rate	Momentum	Decay	Iterations
416 × 416	64	0.001	0.900	0.0005	15,000

**Table 3:** Results for the crack detection of four different methods.

Method	Car	Van	Truck
Artificial	32	6	27
YOLOv4	33	7	27

**Table 4:** Evaluation metrics for the crack detection of three different methods on UCrack.

Method	ODS	OIS	AP
A	0	0	0
B	0.175	0.191	0.073
C	0.614	0.64	0.632

**Table 5:** Evaluation metrics of the six algorithms on the UCrack.

Method	ODS	OIS	AP
DenxiDeepcrack	0.614	0.64	0.632
Deepcrack	0.468	0.51	0.417
Segnet	0.453	0.512	0.405
Unet	0.352	0.413	0.355
Resnet	0.452	0.515	0.413
Fcn	0.351	0.359	0.372

## Data Availability

Not applicable.
